# Quality of Life in Pediatric Oncology Patients in Lebanon (2014-2019): A Multi-center Study

**DOI:** 10.7759/cureus.77000

**Published:** 2025-01-06

**Authors:** Mohamad Ali Hachem, Khoder Al Haj, Mohamad Akil, Bassem Abou Merhi, Nada Sbeity, Mirna N Chahine, Gladys Gemayel, Remy Mckey

**Affiliations:** 1 Hematology and Oncology, Faculty of Medical Sciences, Lebanese University, Beirut, LBN; 2 Obstetrics and Gynecology, Faculty of Medical Sciences, Lebanese University, Beirut, LBN; 3 Emergency Medicine, Faculty of Medical Sciences, Lebanese University, Beirut, LBN; 4 Pediatrics, Makassed General Hospital, Beirut, LBN; 5 Pediatric Oncology, Al-Zahraa Hospital University Medical Center, Beirut, LBN; 6 Basic Sciences, Faculty of Medical Sciences, Lebanese University, Beirut, LBN; 7 Pediatric Oncology, LAU (Lebanese American University) Medical Center - Rizk Hospital, Beirut, LBN; 8 Gastroenterology, Faculty of Medical Sciences, Lebanese University, Beirut, LBN; 9 Internal Medicine, Faculty of Medical Sciences, Lebanese University, Beirut, LBN

**Keywords:** cancer, eortc-qlq-c30, palliative care, pediatrics, quality of life

## Abstract

Aim

Cancer is one of the leading causes of death in pediatrics worldwide. The present study aimed to assess the quality of life (QOL) of pediatric oncology patients diagnosed within the last five years at Lebanese hospitals, and who are residing in Lebanon.

Methods

Pediatric oncology patients were asked to fill out a questionnaire collecting data on personal information, disease status, and QOL using the EORTC-QLQ-C30 (European Organisation for Research and Treatment of Cancer-Quality of Life Questionnaire-Core 30-item version) after receiving their guardian’s consent. IBM SPSS Statistics for Windows, Version 25 (Released 2017; IBM Corp., Armonk, NY, USA) was used for statistical analysis.

Results

The study enrolled 146 patients; 82 (56.2%) were boys. The mean age was 11.39 ± 3.90 years. Significant statistical correlations were found between the mother’s educational level and physical functioning (p-value = 0.006), the number of siblings and each cognitive functioning (p-value = 0.025), and financial difficulties (p-value < 0.0001). The father’s salary was statistically correlated with the patient’s emotional and social functioning and financial difficulties (p-value < 0.05). The type of treatment had a significant effect on cognitive functioning and financial difficulties (p-value < 0.05), with chemotherapy patients having lower scores for financial burden than combination therapy patients. A total of 77.4% (113 patients) had leukemia, 16 (11%) had lymphoma, and 17 (11.6%) had solid tumors. Solid tumor patients had higher cognitive functioning scores than leukemia patients (difference = 12.65 ± 4.74, p-value = 0.023).

Conclusion

Palliative care is needed to relieve the symptom burden and enhance the psycho-social functioning of pediatric oncology patients.

## Introduction

Cancer can develop in individuals of any gender, race, age, and ethnicity, though the rates might vary within those groups [1]. Cancer development is affected by a plethora of genetic and environmental risk factors, whereby the interplay of those factors influences epigenetic mechanisms that can be passed across generations [2]. It is estimated in some studies that genetic factors contribute to no more than 10% of all cancer cases, while lifestyle and environmental factors contribute to the remaining 90% [3].

Among children and adolescents worldwide, cancer is one of the leading causes of death, with an estimated 300,000 children aged between 0 and 19 years diagnosed with cancer annually [4]. The World Health Organization (WHO) reports that the most common cancer types among pediatrics are leukemia, brain cancer, lymphoma, and solid tumors like Wilms tumor and neuroblastoma [5]. Over the last three decades, investments in cancer research have led to the development of effective cancer screening and treatment methods, which have been successful in decreasing death rates and reducing some symptoms and side effects of therapies [6].

Cancer and cancer treatment in children have both positive and negative outcomes after completion. Positive outcomes include superior behavioral conduct, elevated levels of self-worth, and decreased engagement in risky health behaviors [7,8]. On the other hand, cancer has a negative impact on pediatrics, especially because it can disrupt many aspects of their lives, either temporarily or permanently [9]. Some acute symptoms include nausea/vomiting, mouth sores, hair loss, and pain, while chronic problems include obesity, cardiopulmonary dysfunction, second malignancies, sterility, nephrotoxicity, and hepatotoxicity [10]. Surgical procedures often result in deformities, while chemo- and radio-therapy have deleterious side effects and can result in organ dysfunction or late effects in multiple body systems [11]. Knowing that adolescence is the phase where individuals start gaining independence from their parents and focus on building their future academic and work plans, a cancer diagnosis in adolescents often hinders such plans, leaving emotional, cognitive, and social impacts on those diagnosed [12].

The symptoms experienced on a daily basis negatively impact the quality of life (QOL) of cancer patients [13]. Some attempts at increasing the QOL of cancer patients include empowering those individuals by making them active participants in their treatment, aiding distressed families throughout the treatment process, launching follow-up clinics to monitor and support cancer survivors, providing patients with palliative care and psychosocial support services, and encouraging patients to enroll in leisure and creative arts therapies [14-16]. Numerous QOL measuring tools have been launched, such as the EORTC-QLQ-C30 (European Organisation for Research and Treatment of Cancer-Quality of Life Questionnaire-Core 30-item version), which is a self-reported questionnaire consisting of 30 questions to assess the effects of cancer and cancer treatment on the different functions and aspects of cancer patients [17,18].

In Lebanon, there is a lack of published data concerning the assessment of QOL in oncology patients or cancer survivors. A study was conducted in Lebanon in 2013 to assess the QOL of children with cancer; however, a small sample size was enrolled from only one tertiary hospital, and the study only assessed the correlation between patient characteristics and their QOL [19]. As such, more modules should be applied to assess the current welfare of pediatric oncology patients in Lebanon and to identify additional factors. This study aimed to assess the QOL of pediatric cancer patients in Lebanese hospitals, living in Lebanon, and diagnosed in the period from 2014 to 2019.

## Materials and methods

Study design and population

This is an observational, interview-based, analytic, and descriptive questionnaire study considering all pediatric oncology patients diagnosed between 2014 and 2019 in Lebanese hospitals affiliated with the Lebanese University. The study took place from February 1, 2020, until the end of May 2022. The identity of all enrolled patients was kept anonymous. The study included 146 pediatric oncology patients after receiving the consent of their parents or guardians. Patients included in this study were those diagnosed between 2014 and 2019, aged between 5 and 18 years old, and who were conscious, cooperative, and oriented. Excluded patients were those not living in Lebanon, patients with extreme fatal cases, and patients whose guardians did not grant us their consent.

Extreme fatal cases were defined as patients in terminal stages or those receiving end-of-life care. These cases were excluded to ensure that QOL assessments focused on patients undergoing treatment with a reasonable prognosis, as terminal conditions could significantly bias the results.

Data collection

Patients were asked to fill out a questionnaire divided into three sections. Section A required answering some personal questions on gender, age, nationality, total number of siblings, education of the parents, work and salary of the father, and other demographic information. In Section B, patients were asked about their cancer type, duration since diagnosis, treatment type and stage, and remission. Section C consisted of the validated EORTC-QLQ-C30 survey, version 3.0. This questionnaire has a time frame of one week and is filled out using a four-point format ranging from 1 (never) to 4 (always). It consists of 30 questions that are distributed into multiple scales, as presented in Table 1, and scores were obtained following the scoring manual on the EORTC website [20]. Scales were scored over 100: high scores on functional scales and global QOL indicate better health, while high scores on symptom and item scales signify increased burden.

**Table 1:** The distribution of the questions in the EORTC-QLQ-C30 questionnaire into scales. EORTC-QLQ-C30: European Organisation for Research and Treatment of Cancer-Quality of Life Questionnaire-Core 30-item version

Scale		Scale Abbreviation	Number of Questions
Functional Scales	Physical Functioning	PF2	5
	Role Functioning	RF2	2
	Emotional Functioning	EF	4
	Cognitive Functioning	CF	2
	Social Functioning	SF	2
Symptom Scales/Items	Fatigue	FA	3
	Nausea and Vomiting	NV	2
	Pain	PA	2
	Dyspnea	DY	1
	Insomnia	SL	1
	Appetite Loss	AP	1
	Constipation	CO	1
	Diarrhea	DI	1
	Financial Difficulties	FI	1
Global Health Status/Quality of Life		QL2	2

Potential biases from self-reporting by children were mitigated by using age-appropriate, validated questionnaires and providing support for clarification without influencing responses. Parental proxy reporting was also incorporated to complement and validate the children’s self-reports.

Data analysis

Data were analyzed using IBM SPSS Statistics for Windows, Version 25 (Released 2017; IBM Corp., Armonk, NY, USA). Descriptive analysis was conducted using the mean and standard deviation with minimal and maximal values for the quantitative variables, or using frequency and percentage for nominal and ordinal variables. To compare means, analysis of variance (ANOVA) was used, and post-hoc Tukey was then utilized to find significant differences within variables. The independent sample t-test and bivariate correlation (Pearson) were also used for correlation determination for numerical variables. Statistical significance was set at a p-value < 0.05.

Ethical considerations

The study protocol was reviewed and approved by the Institutional Review Board (IRB) at Rafik Hariri University Hospital (RHUH) on January 23, 2020. The confidentiality of the participants was granted, and each participant received a study code.

## Results

Demographic characteristics and family characteristics

Out of the 146 enrolled pediatric oncology patients, 39 were at Zahraa Hospital (26.7%), 54 were at Geitawi Hospital (37%), and the remaining 53 patients were at RHUH (36.3%). Although our study only involves patients residing in Lebanon, 11% of the enrolled patients were non-Lebanese (Iraqis, Palestinians, or Syrians), and the remaining 89% were Lebanese. Concerning gender distribution, 82 patients were male (56.2%) and 64 patients were female (43.8%). The mean age of the patients was 11.39 ± 3.90 years. The patients were mostly between 6 and 11 years old (44.5%), and only 1.4% of the patients were between one and six years old. On average, each patient had three siblings, though some had no siblings, and some had as many as 10 siblings. Moreover, 4.1% of the patients had illiterate mothers, and 2.1% had illiterate fathers. The highest educational level for most mothers was high school (45.2%), while primary education was the most common among fathers (42.5%). Also, 5.5% of the patients had fathers working as healthcare professionals (doctors, nurses, or pharmacists). Law enforcement was the occupation of 15.8% of the patients' fathers (army, security officer, or policeman). Only two patients had unemployed fathers. The most common father’s salary range among the patients was between 675,000 LBP (Lebanese Pound) and 2,280,000 LBP. The detailed distribution of the patients according to their demographics and family characteristics is presented in Table 2.

**Table 2:** Patients demographics and family characteristics according to their parents' level of education as well as the primary provider's occupation and salary (N = 146). LBP: Lebanese Pound

Criteria		Frequency	Percent (%)
Nationality	Lebanese	130	89
	Non-Lebanese	16	11
Gender	Male	82	56.2
	Female	64	43.8
Age Group (Years Old)	1-6	2	1.4
	6-11	65	44.5
	11-16	57	39
	16-21	22	15.1
Mother's Educational Level	Primary Education	44	30.1
	High School	66	45.2
	University	29	19.9
	Technical School	1	0.7
	Illiterate	6	4.1
Father's Educational Level	Primary Education	62	42.5
	High School	51	34.9
	University	26	17.8
	Technical School	4	2.7
	Illiterate	3	2.1
Father's Job	Healthcare	8	5.5
	Engineering	2	1.4
	Teacher	8	5.5
	Law Enforcement	23	15.8
	Transportation	8	5.5
	Other	13	8.9
	Unspecified	82	56.2
	Unemployed	2	1.4
Father's Salary	Less than 675,000 LBP	6	4.1
	Between 675,000 LBP and 2,280,000 LBP	106	72.6
	More than 2,280,000 LBP	10	6.8
	Blank Answer	24	16.4

Cancer and treatment-related characteristics

Out of the 146 patients, 77.4% had leukemia (either acute lymphoblastic leukemia (ALL) or acute myeloid leukemia (AML)), 11% had lymphoma (Hodgkin or non-Hodgkin lymphoma), and the remaining 11.6% reported a solid tumor. Reported solid tumors included Ewing’s sarcoma, glioblastoma, histiocytosis, optic glioma, neurofibromatosis, osteosarcoma, ovarian cancer, rhabdomyosarcoma, hepatoblastoma, and Wilms tumor. The majority of the patients were in remission, having been in remission for an average of 3.38 ± 1.9 years. Patients were either being treated with chemotherapy (88.4%) or with a combination of chemotherapy and either radiotherapy (4.1%), surgery (5.5%), or both surgery and radiotherapy (1.4%). The majority of patients were in the healing stage, and 54.1% had finished treatment. The distribution of patients according to cancer and treatment characteristics is shown in Table 3.

**Table 3:** Clinical data of the enrolled pediatric oncology patients showing that leukemia was the predominant cancer type (77.4%), the majority (72.6%) were in remission, and chemotherapy was the primary treatment modality (88.4%) (N = 146).

Criteria		Frequency	Percent (%)
Cancer Type	Leukemia	113	77.4
	Lymphoma	16	11
	Solid tumors	17	11.6
Remission	Yes	106	72.6
	No	40	27.4
Treatment Type	Chemotherapy	129	88.4
	Chemotherapy + Radiotherapy	6	4.1
	Chemotherapy + Surgery	8	5.5
	Chemotherapy + Radiotherapy + Surgery	2	1.4
	Blank Answer	1	0.7
Treatment Stage	Induction	7	4.8
	Consolidation	4	2.7
	Healing	81	55.5
	Maintenance	41	28.1
	Blank Answers	13	8.9
Finished Treatment	Yes	79	54.1
	No	66	45.2
	Blank Answers	1	0.7

QOL scores

The mean scores of the studied QOL assessment scales varied widely between the enrolled patients, as some patients scored 0 and others 100 on the same scale in the majority of the scales. The highest scores were observed for cognitive functioning (mean CF score = 84.70 ± 18.62) and role functioning (mean RF2 score = 81.85 ± 20.05). The least burdens among symptoms were dyspnea (mean DY score = 11.64 ± 20.56) and constipation (mean CO score = 17.81 ± 20.40). The detailed mean scores of each scale are presented in Table 4.

**Table 4:** Mean scores of the enrolled pediatric oncology patients per each EORTC-QLQ-C30 scale (N = 146 patients). Mean scores for the EORTC-QLQ-C30 scales among the 146 enrolled pediatric oncology patients are presented. The highest scores were observed in cognitive functioning (mean = 84.70) and role functioning (mean = 81.85), while the least burdensome symptoms were dyspnea (mean = 11.64) and constipation (mean = 17.81). This table provides an overview of the variability in quality-of-life measures across patients. EORTC-QLQ-C30: European Organisation for Research and Treatment of Cancer-Quality of Life Questionnaire-Core 30-item version

Scale	Mean Score	Std. Deviation	Minimum	Maximum
PF2 (Physical Functioning)	77.76	20.39	0	100
RF2 (Role Functioning)	81.85	20.05	0	100
EF (Emotional Functioning)	77.68	18.80	16.67	100
CF (Cognitive Functioning)	84.70	18.62	16.67	100
SF (Social Functioning)	74.09	25.82	0	100
FA (Fatigue)	21.00	20.09	0	88.89
NV (Nausea and Vomiting)	24.32	22.33	0	66.67
PA (Pain)	22.03	21.79	0	100
DY (Dyspnea)	11.64	20.56	0	66.67
SL (Insomnia)	19.18	24.71	0	100
AP (Appetite Loss)	23.52	26.60	0	100
CO (Constipation)	17.81	20.40	0	66.67
DI (Diarrhea)	19.63	23.05	0	100
FI (Financial Difficulties)	46.80	35.14	0	100
QL2 (Global Health Status/Quality of Life)	76.94	20.83	16.67	100

Factors affecting QOL

Type of Cancer

While assessing psychological distress according to the type of cancer, the one-way ANOVA results showed only a statistically significant difference in cognitive functioning between the mean scores of patients according to cancer type (p-value = 0.017). The post-hoc tests for multiple comparisons indicated that only the variation in cognitive functioning scores between leukemia and solid tumors was statistically significant (p-value = 0.023), with patients with solid tumors having higher cognitive functioning scores than leukemia patients (average score difference of 12.65 ± 4.74). No other statistically significant score differences were observed in social and emotional functioning scores (p-value > 0.05), as presented in Table 5.

**Table 5:** Variation in emotional, cognitive, and social functioning scores according to cancer type. One-way ANOVA was used to assess differences in functioning scores (emotional, cognitive, and social) across cancer types (leukemia, lymphoma, and solid tumors). Post-hoc multiple comparisons were performed using Tukey’s test to identify specific group differences. Statistically significant differences (p < 0.05) are indicated with an asterisk (*). Cognitive functioning scores showed a statistically significant difference between patients with leukemia and those with solid tumors (p = 0.023), with solid tumor patients scoring higher by an average of 12.65 points. No significant differences were observed in emotional or social functioning scores across cancer types. ANOVA: Analysis of variance

Dependent Variable	Cancer Type (I)	Cancer Type (J)	Mean Difference (I-J)	Significant
					EF (Emotional Functioning)	Leukemia	Lymphoma	-9.32	0.15
Solid Tumors	4.1	0.675				
Lymphoma	Leukemia	9.32	0.15		
	Solid Tumors	13.42	0.1		
Solid Tumors	Leukemia	-4.1	0.675		
	Lymphoma	-13.42	0.1		
CF (Cognitive Functioning)	Leukemia	Lymphoma	-7.13	0.31
		Solid Tumors	-12.65*	0.023
	Lymphoma	Leukemia	7.13	0.31
		Solid Tumors	-5.51	0.661
	Solid Tumors	Leukemia	12.65*	0.023
		Lymphoma	5.51	0.661
SF (Social Functioning)	Leukemia	Lymphoma	-10.17	0.304
		Solid Tumors	-7.29	0.523
	Lymphoma	Leukemia	10.17	0.304
		Solid Tumors	2.88	0.945
	Solid Tumors	Leukemia	7.29	0.523
		Lymphoma	-2.88	0.945

Number of Siblings

Upon checking for correlations between the number of siblings and each of the functional scales, financial difficulties, and global QOL, only significant correlations were found with cognitive functioning (p-value = 0.025) and financial difficulties (p-value < 0.0001), as shown in Table 6.

**Table 6:** Correlation between number of siblings and each of the functional scales scores, financial difficulties score, and global health status score. Pearson’s correlation coefficient was used to assess the relationship between the number of siblings and the functional scales, financial difficulties, and global QOL scores. Statistically significant correlations are indicated as follows: *p < 0.05; **p < 0.001. Significant positive correlations were observed with cognitive functioning (p = 0.025) and financial difficulties (p < 0.001), indicating that a higher number of siblings is associated with better cognitive functioning and increased financial difficulties. QOL: Quality of life

Correlations		PF2 (Physical Functioning)	RF2 (Role Functioning)	EF (Emotional Functioning)	CF (Cognitive Functioning)	SF (Social Functioning)	FI (Financial Difficulties)	QL2 (Global Health Status/Quality of Life)
Siblings	Pearson Correlation	0.025	0.084	-0.083	0.185*	0.052	0.399**	0.139
	Significant (Two-Tailed)	0.765	0.311	0.318	0.025	0.53	0	0.095
	N	146	146	146	146	146	146	146

Cancer Treatment Type

The applied one-way ANOVA test between all QOL scales in the questionnaire and cancer treatment type showed that statistically significant differences were only observed in cognitive functioning (p-value = 0.047) and financial difficulties (p-value = 0.045). Patients undergoing chemotherapy had significantly less financial burden than those undergoing both chemotherapy and radiotherapy (mean score difference of 39.15 points, p-value = 0.0036). Surprisingly, no significant mean difference was observed in the cognitive functioning scores between patients of different treatment modalities, possibly due to the discrepancy in the number of patients undergoing each treatment type. Table 7 only presents statistically significant results.

**Table 7:** Mean score differences in cognitive functioning and financial difficulties according to treatment type. One-way ANOVA was used to evaluate differences in cognitive functioning and financial difficulties scores across cancer treatment types. Post-hoc multiple comparisons (e.g., Tukey's test) were performed to identify significant pairwise differences. Statistically significant differences (p < 0.05) are marked with an asterisk (*). Significant differences in financial difficulties scores were observed among treatment types, with chemotherapy-only patients experiencing less financial burden compared to those undergoing both chemotherapy and radiotherapy. Cognitive functioning differences were not statistically significant across treatment groups. ANOVA: Analysis of variance

Dependent Variable	Treatment Type (I)	Treatment Type (J)	Mean Difference (I-J)	Significant
CF (Cognitive Functioning)	Chemotherapy	Chemotherapy + Radiotherapy	-14.15	0.255
		Chemotherapy + Surgery	-12.76	0.228
		Chemotherapy + Radiotherapy + Surgery	-16.93	0.567
	Chemotherapy + Radiotherapy	Chemotherapy	14.15	0.255
		Chemotherapy + Surgery	1.39	0.999
		Chemotherapy + Radiotherapy + Surgery	-2.78	0.998
	Chemotherapy + Surgery	Chemotherapy	12.76	0.228
		Chemotherapy + Radiotherapy	-1.39	0.999
		Chemotherapy + Radiotherapy + Surgery	-4.17	0.992
	Chemotherapy + Radiotherapy + Surgery	Chemotherapy	16.93	0.567
		Chemotherapy + Radiotherapy	2.78	0.998
		Chemotherapy + Surgery	4.17	0.992
FI (Financial Difficulties)	Chemotherapy	Chemotherapy + Radiotherapy	-39.15*	0.036
		Chemotherapy + Surgery	-5.81	0.967
		Chemotherapy + Radiotherapy + Surgery	-22.48	0.795
	Chemotherapy + Radiotherapy	Chemotherapy	39.15*	0.036
		Chemotherapy + Surgery	33.33	0.279
		Chemotherapy + Radiotherapy + Surgery	16.67	0.934
	Chemotherapy + Surgery	Chemotherapy	5.81	0.967
		Chemotherapy + Radiotherapy	-33.33	0.279
		Chemotherapy + Radiotherapy + Surgery	-16.67	0.928
	Chemotherapy + Radiotherapy + Surgery	Chemotherapy	22.48	0.795
		Chemotherapy + Radiotherapy	-16.67	0.934
		Chemotherapy + Surgery	16.67	0.928

Age at Diagnosis

The correlation of age at diagnosis with the severity of each of the symptoms was assessed; however, bivariate correlation results showed no significant correlation with any. It is important to note that correlation tests were applied to other characteristics, such as gender and age of the patients, but no statistically significant results were obtained (all p-values were > 0.05), signifying no statistical correlation between any of the studied scale scores and either gender or age.

Financial Status

Upon checking the correlation between the father’s salary and psychological distress, significance was only found in emotional and social functioning (p-values = 0.009 and 0.016, respectively). A positive correlation was also found between father’s salary and financial difficulties (p-value = 0.005). Patients whose father’s salary was less than 675,000 LBP had significantly lower emotional functioning scores and higher financial difficulty scores compared to other patients (p-values < 0.05). Patients whose father’s salary was less than 675,000 LBP had significantly lower social functioning scores than patients whose fathers were paid between 675,000 LBP and 2,280,000 LBP (p-value = 0.012). The mean differences and significance of these studied factors are presented in Table 8.

**Table 8:** Mean score differences in emotional functioning, social functioning, and financial difficulties according to father’s salary. One-way ANOVA was used to analyze differences in emotional functioning, social functioning, and financial difficulties scores among groups based on the father’s salary. Post-hoc multiple comparisons (e.g., Tukey's test) were conducted to identify significant pairwise differences. Statistically significant differences (p < 0.05) are marked with an asterisk (*). Significant correlations were found between the father’s salary and emotional functioning, social functioning, and financial difficulties. Patients with fathers earning less than 675,000 LBP reported worse emotional and social functioning, and greater financial difficulties compared to higher-income groups. ANOVA: Analysis of variance; LBP: Lebanese Pound

Dependent Variable	Salary of the Father (I)	Salary of the Father (J)	Mean Difference (I-J)	Significant
					EF (Emotional Functioning)	Less than 675,000 LBP	Between 675,000 LBP and 2,280,000 LBP	-23.37*	0.008
More than 2,280,000 LBP	-26.11*	0.018				
Between 675,000 LBP and 2,280,000 LBP	Less than 675,000 LBP	23.37*	0.008		
	More than 2,280,000 LBP	-2.74	0.893		
More than 2,280,000 LBP	Less than 675,000 LBP	26.11*	0.018		
	Between 675,000 LBP and 2,280,000 LBP	2.74	0.893		
SF (Social Functioning)	Less than 675,000 LBP	Between 675,000 LBP and 2,280,000 LBP	-30.08*	0.012
		More than 2,280,000 LBP	-28.89	0.063
	Between 675,000 LBP and 2,280,000 LBP	Less than 675,000 LBP	30.08*	0.012
		More than 2,280,000 LBP	1.19	0.988
	More than 2,280,000 LBP	Less than 675,000 LBP	28.89	0.063
		Between 675,000 LBP and 2,280,000 LBP	-1.19	0.988
FI (Financial Difficulties)	Less than 675,000 LBP	Between 675,000 LBP and 2,280,000 LBP	38.99*	0.016
		More than 2,280,000 LBP	56.67*	0.003
	Between 675,000 LBP and 2,280,000 LBP	Less than 675,000 LBP	-38.99*	0.016
		More than 2,280,000 LBP	17.67	0.241
	More than 2,280,000 LBP	Less than 675,000 LBP	-56.67*	0.003
		Between 675,000 LBP and 2,280,000 LBP	-17.67	0.241

Parent’s Educational Level

For studying the effect of parents' educational level on coping with the illness, scores for all scales except symptoms and financial difficulties were compared. No statistically significant correlation was found between any of the scales and the father’s education. The only scale showing significance was physical functioning (p-value = 0.039), where patients whose mothers had university degrees had significantly higher mean physical functioning scores than those whose mothers were only high school graduates (72.83 versus 85.98, respectively; p-value = 0.006).

## Discussion

This study assessed the QOL of 146 pediatric oncology patients diagnosed within the last five years and treated in three hospitals affiliated with the Lebanese University. By including multiple hospitals and oncologists, we aimed to mitigate potential biases arising from specific institutional or physician practices, ensuring more generalizable results. The cohort’s demographic characteristics reflect the Lebanese population, with 56.2% of participants being boys, a mean age of 11.39 ± 3.90 years, and 89% identifying as Lebanese. The inclusion of patients from other nationalities (11%) aligns with the refugee influx to Lebanon, as previously reported in similar studies [21].

Demographic characteristics, such as the number of siblings, parental education, and father’s salary, were chosen due to their potential influence on QOL outcomes. The average number of siblings (four per patient) positively correlated with cognitive functioning (p = 0.025) and financial difficulties (p < 0.0001). Larger families often provide emotional support, which can enhance cognitive functioning [22,23], but simultaneously increase financial strain, highlighting the dual impact of family dynamics on QOL.

Educational attainment also played a critical role. Mother’s education significantly correlated with cognitive functioning (p < 0.05), emphasizing the influence of maternal involvement and awareness on the psychological well-being of children. These findings underscore the necessity of targeted educational interventions to enhance parental understanding of cancer care and coping mechanisms.

Socioeconomic status, measured through the father’s salary, had significant effects on emotional and social functioning (p = 0.009 and 0.016, respectively) and financial difficulties (p = 0.005). Patients with fathers earning below the Lebanese minimum wage (675,000 LBP) reported lower emotional and social functioning scores and higher financial difficulties [24]. This underscores the critical need for targeted financial support programs for low-income families to alleviate the economic burden and improve patient outcomes [24,25].

Comparisons with global and regional studies further contextualize these findings. Our functional and symptom scores were generally lower than those reported by Derogar et al. in Sweden [26], reflecting the economic challenges and healthcare disparities in Lebanon. Notably, financial difficulties were markedly higher in our population (46.80 vs. 4.4), consistent with Lebanon’s ongoing economic crisis. However, comparisons with regional studies - such as those from other Middle Eastern countries experiencing similar economic pressures - would provide a more nuanced understanding of these disparities. For instance, studies in Egypt and Jordan have highlighted similar challenges in financial and emotional functioning among pediatric cancer patients [27,28].

Cancer type and treatment modalities also influenced QOL outcomes. The majority (77.4%) of patients had leukemia, consistent with global trends [29]. While cognitive functioning was significantly lower in leukemia patients compared to those with solid tumors (p = 0.023), no significant differences were observed in other QOL scales, suggesting that disease-specific interventions may be required. Treatment type influenced financial difficulties, with chemotherapy alone associated with lower financial strain compared to combination therapies (p = 0.036). These findings suggest that cost-effective treatment strategies could alleviate financial burdens while maintaining therapeutic efficacy.

To address these findings, actionable recommendations include implementing financial aid programs for low-income families, integrating routine cognitive and emotional assessments into pediatric oncology care, and providing parental education workshops. Psychological interventions, such as family counseling and peer support groups, can further mitigate the emotional and social challenges identified in this study. Policymakers should prioritize these measures to enhance holistic care in pediatric oncology.

Study limitations

This was a descriptive observational questionnaire-based study on self-reported data from pediatric oncology patients. This permits bias in reporting the actual severity of the symptoms and psychological distress. Some answers were also left unanswered, which could have been either by mistake or because the children did not know the answers to these questions. The study was conducted in only three hospitals, but larger studies, including more centers and possibly from different countries, are needed to eliminate possible bias. Moreover, the study design does not allow for determining actual causality; it only provides possible correlations and the strength of the association between QOL scores and the studied factors.

## Conclusions

This study highlights the multifaceted impact of socioeconomic and demographic factors on the QOL of pediatric oncology patients in Lebanon. Financial strain, family dynamics, and parental education emerged as significant determinants, underscoring the need for targeted support systems. Policies should focus on providing financial assistance, enhancing parental education, and incorporating psychological interventions into standard care protocols. Future research should explore regional comparisons and longitudinal assessments to better inform policy and clinical practice. By addressing these challenges, healthcare systems can improve the overall well-being and outcomes of pediatric oncology patients.
